# Prediction of breast cancer molecular subtypes using radiomics signatures of synthetic mammography from digital breast tomosynthesis

**DOI:** 10.1038/s41598-020-78681-9

**Published:** 2020-12-09

**Authors:** Jinwoo Son, Si Eun Lee, Eun-Kyung Kim, Sungwon Kim

**Affiliations:** Department of Radiology, Research Institute of Radiological Science and Center for Clinical Image Data Science, Severance Hospital, Yonsei University College of Medicine, 50-1 Yonsei-ro, Seodaemun-gu, Seoul, 03722 Republic of Korea

**Keywords:** Breast cancer, Cancer imaging

## Abstract

We aimed to predict molecular subtypes of breast cancer using radiomics signatures extracted from synthetic mammography reconstructed from digital breast tomosynthesis (DBT). A total of 365 patients with invasive breast cancer with three different molecular subtypes (luminal A + B, luminal; HER2-positive, HER2; triple-negative, TN) were assigned to the training set and temporally independent validation cohort. A total of 129 radiomics features were extracted from synthetic mammograms. The radiomics signature was built using the elastic-net approach. Clinical features included patient age, lesion size and image features assessed by radiologists. In the validation cohort, the radiomics signature yielded an AUC of 0.838, 0.556, and 0.645 for the TN, HER2 and luminal subtypes, respectively. In a multivariate analysis, the radiomics signature was the only independent predictor of the molecular subtype. The combination of the radiomics signature and clinical features showed significantly higher AUC values than clinical features only for distinguishing the TN subtype. In conclusion, the radiomics signature showed high performance for distinguishing TN breast cancer. Radiomics signatures may serve as biomarkers for TN breast cancer and may help to determine the direction of treatment for these patients.

## Introduction

Breast cancer is the most common cancer diagnosed in women, and the second leading cause of all cancer-related deaths^1^. Early diagnosis of breast cancer and prediction of prognosis are the key goals of current clinical research.

Depending on the expression level of certain receptors, breast cancer can be divided into various subtypes, such as the luminal, human epidermal growth factor receptor 2 (HER2)-enriched, and triple-negative (TN) subtype^2,3^. Among these, cancers of the TN subtype are more aggressive and difficult to treat^2,4,5^. Therefore, they account for a large portion of breast cancer deaths that occur after diagnosis^6^. Patients with TN breast cancer derive no benefit from endocrine therapy or trastuzumab, because they lack the appropriate targets for these drugs. On the other hand, TN breast cancer responds well to neoadjuvant chemotherapy and patients with good response show improved prognosis^7–9^.

Several reports have found that findings on mammography, ultrasonography, or MRI are related to the molecular subtypes of breast cancer^10–12^. Recently, several attempts have been made to predict these molecular subtypes through a radiomics approach. Radiomics refers to the transformation of image data into computer-based, high-dimensional data. The resulting data reflect not only tissue characteristics but also gene expression^13^. A few studies have shown that radiomics features obtained from magnetic resonance imaging (MRI) can be associated with the molecular subtypes of breast cancer^14–16^.

Mammography is the primary modality for breast cancer diagnosis and can be performed in all patients while being highly accessible. Although MRI has advantages in tissue characterization, it is not yet a routine modality for all patients. Therefore, being able to predict molecular subtype by routinely performed mammography will be of clinical value, and several previous studies have shown the possibilities^17,18^.

The use of digital breast tomosynthesis (DBT) has increased, and adding DBT to digital mammography can increase the detection rate in breast cancer screening over digital mammography alone^19,20^. However, using DBT with digital mammography for screening also increases the radiation dose^21^. To overcome this, a method was developed to reconstruct synthetic mammography images from information acquired during a DBT data acquisition. More and more evidence indicates that synthetic mammography will eventually be able to replace digital mammography^22,23^. Despite DBT becoming the primary modality in breast cancer diagnosis, there are problems such as higher reading workload^24^ and inconsistency in mass segmentation, owing to the numerous slices of images. This limits the practicality and reproducibility in applying radiomics to DBT. Therefore, synthetic mammography can be a good methodology for applying radiomics in clinical practice. However, to our knowledge, there is no research on using the radiomics approach on synthetic mammography from DBT for molecular subtyping.

The purpose of this study was to investigate whether radiomics features obtained from synthetic mammography images reconstructed from DBT can distinguish different molecular subtypes of breast cancer.

## Methods

### Patient selection

This retrospective study was approved by the Institutional Review Board of Severance Hospital in Seoul, Korea. The requirement for informed consent was waived. All methods described in this manuscript were performed in accordance with the approved guidelines and regulations.

From December 2015 to September 2016, 691 patients who were pathologically diagnosed with invasive breast cancer and had preoperative DBT were enrolled in this study. Exclusion criteria were: (1) patients who received chemotherapy before DBT (n = 114), (2) patients who received surgical excision or vacuum-assisted biopsy (n = 41), (3) asymmetries that were only visible on a single view (n = 40), (4) diffuse infiltrative lesions involving the whole breast (n = 7), (5) lesions partially masked by a marker (n = 15), (6) lesions not fully included on synthetic mammography (n = 34), and (7) lesions not clearly delineated on synthetic mammography (n = 75).

**Figure 1:**
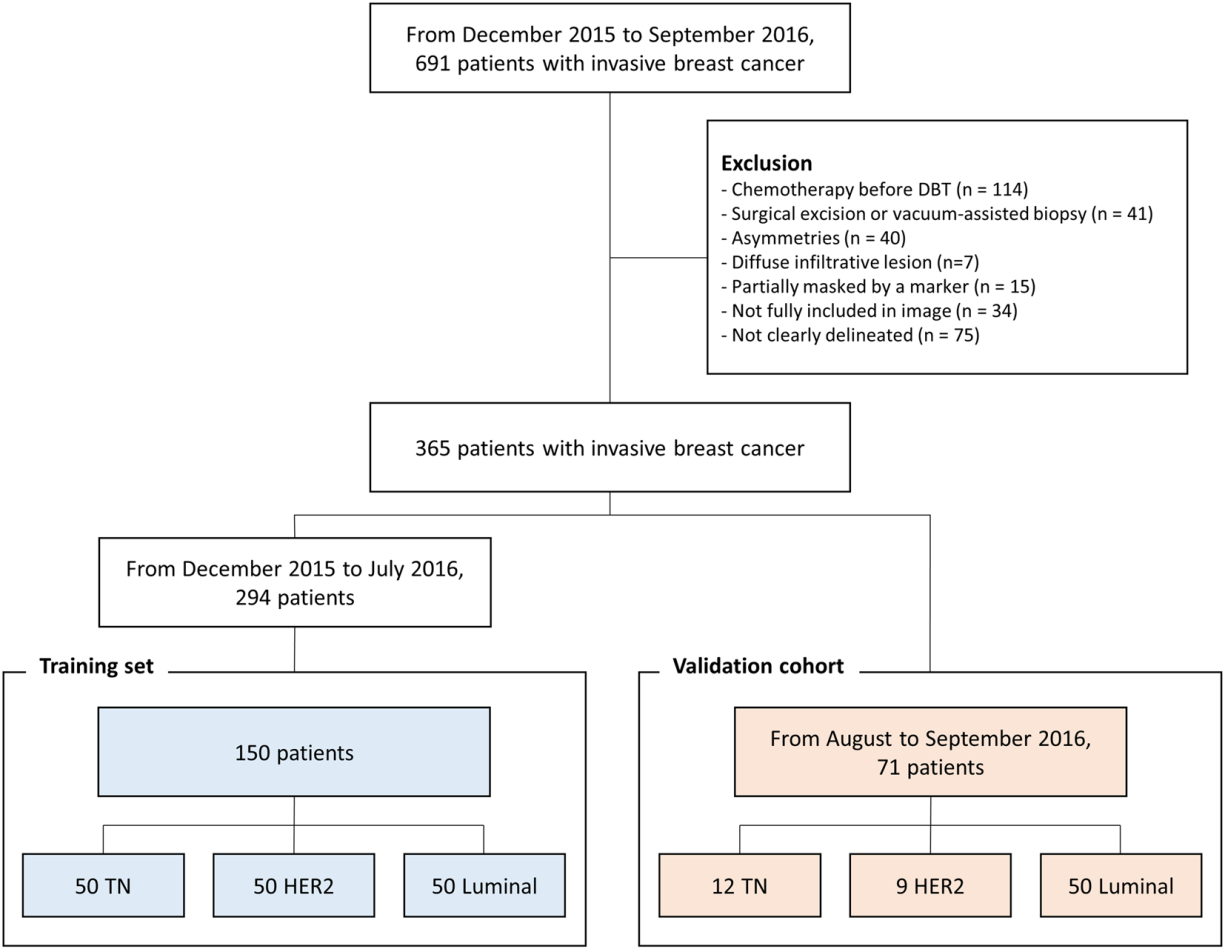
Patient selection.

Finally, 365 patients were included in this study. Because there are remarkable differences in incidence among molecular subtypes^25^, the same number of patients was assigned to each group to avoid inappropriate feature selection due to class imbalance and to improve the performance of classification^26,27^. Among the 294 patients who were diagnosed with breast cancer between December 2015 and July 2016, 50 consecutive patients were selected for each molecular subtype and assigned to the training set. The remaining cases were not included in the analysis. Accordingly, a total of 150 patients were finally included in the training set. For the validation cohort, 71 temporally independent patients who were diagnosed with breast cancer between August 2016 and September 2016 were included. The composition of the temporal validation was done according to the transparent reporting of a multivariable prediction model for individual prognosis or diagnosis (TRIPOD) statement^28^. The validation cohort consisted of 50 patients of the luminal subtype, 9 of the HER2 subtype and 12 of the TN subtype (Fig. 1).

### Pathologic examination

Pathologic diagnoses were based on postoperative tissue samples. A pathologic report of all breast cancers included the expression levels of the estrogen receptor (ER), progesterone receptor (PR) and HER2. Breast cancers were classified as “Luminal”, “HER2 (HER2-enriched)” or “TN (triple negative)” according to the ASCO/CAP guidelines^29^. In our study, the luminal subtype included luminal A and luminal B. ER and/or PR positive breast cancers were classified as the luminal subtype. ER and PR negative with HER2 positive breast cancers were classified as the HER2-enriched subtype. ER, PR, and HER2 negative breast cancers were classified as the TN subtype. For ER and PR, more than 1% of expression indicated positivity. For HER2, 3 + indicated positivity. For equivocal expression of HER2 (2 +), fluorescence in situ hybridization (FISH) 2.0 or higher indicated positivity.

### Image acquisition & tumor segmentation

DBT was performed using a mammography machine (Selenia Dimensions System; Hologic,Marlborough, MA) with bilateral craniocaudal (CC) and mediolateral oblique (MLO) views. The X-ray tube rotated in a 15° arc with the breast compressed, and there were 15 projections for each view. After scanning, the projection data from the frames were combined to create 3D DBT images, and 2D synthetic mammography images were concurrently processed. In-plane resolution of synthetic mammography was 1890 × 2457 pixels for both MLO and CC views.

All synthetic mammography images underwent the following preprocessing steps before the radiomics analysis. Each pixel was resampled to 0.1 × 0.1 mm in size, because this might affect radiomics features related to spatial information or tumor texture^30^. The intensities of the pixels covering the breast were adjusted to have the same mean and standard deviation for all images.

**Figure 2:**
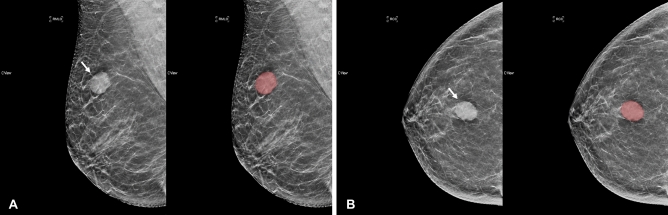
Segmentation example 1. Example of tumor segmentation on synthetic mammography. The synthetic mediolateral oblique **(A)** and craniocaudal **(B)** views of a 58-year-old female diagnosed with the triple negative subtype of breast cancer. The breast lesion appears as a circumscribed and round mass with high density (arrow).

**Figure 3:**
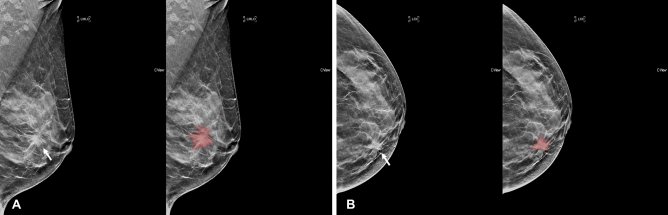
Segmentation example 2. Example of tumor segmentation on synthetic mammography. The synthetic mediolateral oblique **(A)** and craniocaudal **(B)** views of a 47-year-old female diagnosed with the luminal subtype of breast cancer. The breast lesion appears as a spiculated mass with architectural distortion (arrow).

The 2D region of interest (ROI) covering the tumor on synthetic mammography was manually segmented (Figs. 2 and 3) by one resident radiologist with 3 years of experience (reader 1) using the “MIPAV” software (https://mipav.cit.nih.gov). Then, the drawn ROIs were checked in detail and confirmed by a breast radiologist with 25 years of subspecialty experience (reader 2). Disagreements about the ROI were resolved by a consensus-based discussion. Another breast radiologist with 1 year of subspecialty experience (reader 3) independently drew ROIs on images for 40 randomly selected patients from the training set to evaluate interobserver reproducibility. All readers were blinded to the molecular subtype or the pathologic report of the breast cancer.

### Radiomics feature extraction & selection

Radiomics features were calculated based on segmented ROIs using an open source software, “PyRadiomics” (https://pyradiomics.readthedocs.io, version 2.1.2)^31^. The categories of the radiomics features were as follows: (1) first order; 18 features, (2) GLCM; 22 features, (3) GLRLM; 16 features, and (4) GLSZM; 16 features. A full list of the features included in each category is described in the supplementary materials (Supplementary Table 1). Image filters such as Laplacian of Gaussian or wavelets were not used in this study for a more intrinsic interpretation of the radiomics features. A total of 72 radiomics features were obtained for each view.

**Table 1:** Characteristics of patients and lesions.

	TN			HER2			Luminal		
	Training set (N = 50)	Validation set (N = 12)	P value	Training set (N = 50)	Validation set (N = 9)	P value	Training set (N = 50)	Validation set (N = 50)	P value
Age*	54.08 ± 10.48	51.08 ± 11.80	0.388	52.70 ± 8.51	53.22 ± 12.85	0.877	55.66 ± 10.95	50.36 ± 12.58	0.027
Lesion size (mm)*	33.98 ± 17.45	29.47 ± 15.04	0.894	41.78 ± 19.55	28.33 ± 10.17	0.050	24.92 ± 14.41	28.68 ± 14.12	0.191
Menopausal status			0.841			0.861			0.104
Premenopausal	12	2		12	2		12	22	
Postmenopausal	35	9		33	7		36	25	
Not reported	3	1		5	0		2	3	
Invasive cancer			1			1			0.617
Ductal	50	12		50	9		47	49	
Lobular	0	0		0	0		3	1	
LN status			0.990			0.431			0.837
Positive	11	2		11	3		20	18	
Negative	39	10		39	6		30	32	

*TN* triple-negative, *LN* lymph node.

* Data are means ± standard deviations.

The elastic-net approach was used to select appropriate features and to build the radiomics model. Elastic-net is a logistic regression model which combines ridge regression and the least absolute shrinkage and selection operator (LASSO)^32,33^. Parameter tuning of the elastic-net was performed through ten-fold cross-validation. For the tuning coefficients λ and α, the criterion of minimum standard deviation and maximum AUC were applied, respectively.

Feature selection and modeling processes were done in the training set, using R software (version 3.5.1; http://www.Rproject.org)^34^ and the “glmnet” package (version 2.0–16)^33^.

### Molecular subtype classification

We performed three binary classifications to predict molecular subtypes. This was to obtain intuitive results while avoiding statistical complexity^17,35^. In order to overcome the imbalanced number of lesions belonging to each category in the modeling process, we applied the synthetic minority oversampling technique (SMOTE) method. SMOTE is an oversampling method that is commonly used to improve random oversampling^36,37^. After the modeling process, selected features were extracted and their linear combinations formed the radiomics signature of each lesion.

The modeling process was repeated for features obtained from the CC view only (CC model), features obtained from the MLO view only (MLO model), and concatenated features obtained from both views (CC + MLO model).

### Clinical feature assessment

For all breast cancer lesions, two radiologists (reader 1 and reader 2) evaluated the lesions on synthetic mammography images based on the Breast Imaging Reporting and Data System (BI-RADS)^38^. When the two radiologists made different observations, a consensus was reached for the final assessment. Clinical features in this study were patient age, lesion size and mammographic features based on BI-RADS.

### Statistical analysis

Continuous values were compared with the Student's t-test. All continuous variables were verified for normality by the Shapiro–Wilk test. Categorical variables were compared with Pearson's Chi-square test or Fisher's exact test. Univariate and multivariate logistic regression analyses for clinical features were done to identify independent predictors of the molecular subtypes of breast cancer. A “combined model” was built by performing multivariate logistic regression that included both the radiomics signature and the independent variables from the multivariate analysis of clinical features. A two-sided P < 0.05 was considered to indicate a statistically significant difference. Classification performances were evaluated based on the receiver operating characteristic (ROC) curve and area under the curve (AUC) in the validation cohort. The two ROC curves were compared using Delong's test. Consistency of the predicted and actual probabilities of a model was demonstrated by a calibration curve. To assess the clinical usefulness of a model, decision curve analysis was used to quantify the net benefit at different threshold probabilities in the validation cohort. The radiomics signature and the BI-RADS features were correlated using Pearson’s correlation coefficient. Interobserver reproducibility was assessed with the intraclass correlation coefficient (ICC). An ICC > 0.75 was considered to indicate good agreement.

## Results

A total of 150 patients (TN = 50, HER2 = 50, Luminal = 50) were assigned to the training set and 71 patients (TN = 12, HER2 = 9, Luminal = 50) were assigned to the validation cohort (Table 1, Supplementary Table 2). All continuous variables showed normal distribution.

**Table 2:** Classification performance of the radiomics models in the validation cohort.

		TN vs non-TN	HER2 vs non-HER2	Luminal vs non-luminal
CC model	AUC	0.819	0.520	0.659
	Accuracy	0.817	0.761	0.563
	Sensitivity	0.750	0.222	0.440
	Specificity	0.831	0.839	0.867
MLO model	AUC	0.791	0.645	0.627
	Accuracy	0.718	0.747	0.521
	Sensitivity	0.917	0.111	0.480
	Specificity	0.678	0.839	0.619
CC + MLO model	AUC	0.838	0.556	0.645
	Accuracy	0.803	0.704	0.507
	Sensitivity	0.833	0.111	0.440
	Specificity	0.797	0.790	0.667

*TN* triple-negative, *AUC* area under the receiver operating characteristic curve.

### Radiomics features and prediction performance

Among all the radiomics features, 71 features in the MLO view and 58 features in the CC view showed good interobserver reproducibility (Supplementary Fig. 1). Finally, a total of 129 features were included in the analysis.

When concatenating (CC + MLO model) all features, 20 features were selected for TN vs non-TN, 18 for HER2 vs non-HER2, and 66 features for luminal vs non-luminal. A list of the selected features is included in the supplementary materials (Supplementary Table 3). When only features from the CC view were included (CC model), 6 features were selected for TN vs non-TN, 34 for HER2 vs non-HER2, and 43 features for luminal vs non-luminal. For the MLO view (MLO model), 17 features were selected for TN vs non-TN, 34 for HER2 vs non-HER2, and 42 features for luminal vs non-luminal. In the training set, the CC + MLO model yielded an AUC of 0.834 for TN, 0.842 for HER2, and 0.941 for the luminal subtype.

**Table 3:** Comparison of AUC (area under the receiver operating characteristic curve) values between the radiomics models (P value) with Delong’s test.

	TN vs non-TN	HER2 vs non-HER2	Luminal vs non-luminal
CC model vs MLO model	0.646	0.354	0.544
CC model vs CC + MLO model	0.526	0.694	0.742
MLO model vs CC + MLO model	0.250	0.171	0.647

In the validation cohort, the CC + MLO model yielded an AUC of 0.838 for TN, 0.556 for HER2, and 0.645 for the luminal subtype. With the optimal cut-off value of the radiomics signature was applied in this model, the sensitivity and specificity of the models in the validation cohort were 83.3% and 79.7% for TN, 11.1% and 79.0% for HER2, 44.0% and 66.7% for the luminal subtype, respectively (Table 2).

When the AUCs of the CC + MLO, CC and MLO models were compared for the three binary classifications, no statistically significant differences were found (Table 3).

### Comparison of prediction performance between the clinical and the radiomics models

We compared the predictive performance of the clinical model with the combined model. In the TN subtype, the univariate analysis of the clinical features showed that round shape, high density and architectural distortion were statistically significant features. In the multivariate analysis of the clinical model, round shape and high density were identified as independent factors for predicting the TN subtype (Table 4). The clinical model showed an AUC of 0.665 in the validation cohort (Table 5).

**Table 4:** Univariate and multivariate logistic regression of the clinical model and combined model for the TN subtype of breast cancer.

Feature	TN	Non-TN	Univariate analysis		Multivariate analysis		With radiomics signature	
			P value	Odds ratio	P value	Odds ratio	P value	Odds ratio
Age	54.08 ± 10.48	54.18 ± 9.870	0.954	0.999 (0.965, 1.034)				
Size	33.98 ± 17.45	33.35 ± 19.07	0.844	1.002 (0.983, 1.020)				
**Breast composition**								
Dense Fatty	40 10	71 29	Ref 0.239	1 0.612 (0.260, 1.351)				
**Gross feature**								
Mass only Mass + calcification Calcification only	29 21 0	46 47 7	Ref 0.330 0.986	1 0.709 (0.351, 1.413) NA				
**Shape**								
Oval Round Irregular	3 17 30	4 12 77	0.409 0.003 Ref	1.925 (0.362, 9.235) 3.636 (1.567, 8.696) 1	0.016	3.028 (1.233, 7.681)	0.335	1.695 (0.575, 4.998)
**Mass margin**								
Obscured Microlobulated Indistinct Spiculated	10 7 28 5	18 9 50 16	0.986 0.555 Ref 0.301	0.992 (0.393, 2.414) 1.389 (0.452, 4.134) 1 0.558 (0.168, 1.598)				
**Mass density**								
Low Equal High	3 23 24	7 61 24	0.861 Ref 0.013	1.137 (0.230, 4.478) 1 2.546 (1.223, 5.372)	0.018	2.542 (1.180, 5.573)	0.370	1.525 (0.598, 3.834)
Architectural distortion	5	25	0.036	0.333 (0.107, 0.869)	0.107	0.403 (0.121, 1.143)	0.419	0.575 (0.138, 2.084)
**Calcification morphology**								
Benign Amorphous Coarse heterogeneous Fine pleomorphic Fine linear branching	1 2 3 11 4	1 2 5 33 14	0.451 0.300 0.468 Ref 0.817	3.000 (0.112, 80.288) 3.000 (0.329, 27.556) 1.800 (0.327, 8.635) 1 0.857 (0.209, 3.009)				
**Calcification distribution**								
Diffuse Regional Grouped Linear Segmental	0 2 4 1 14	1 2 13 0 39	0.992 0.328 0.813 0.991 Ref	NA 2.786 (0.310, 25.086) 0.857 (0.214, 2.907) NA 1				
Radiomics signature			< 0.001	1781 (190, 23,225)			< 0.001	828 (78, 12,147)

The 95% confidence intervals of the AUCs are shown in parentheses.

*TN* triple-negative, *AUC* area under the receiver operating characteristic curve.

**Table 5:** AUC (area under the receiver operating characteristic curve) values of the clinical and combined model in the validation cohort.

	Clinical model	Combined model	P value
TN	0.665 (0.504–0.826)	0.868 (0.730–1.000)	0.045
HER2	0.501 (0.230–0.771)	0.582 (0.361–0.804)	0.159
Luminal	0.680 (0.554–0.806)	0.677 (0.552–0.802)	0.952

The 95% confidence intervals of the AUCs are shown in parentheses.

*TN* triple-negative.

**Figure 4:**
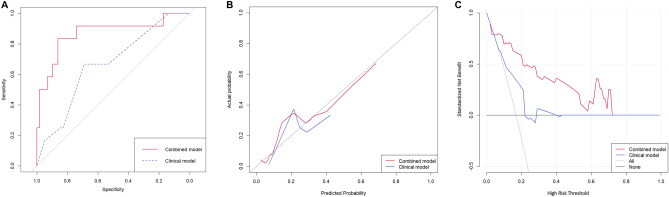
The ROC curve, calibration curve and decision curve of clinical and combined models for distinguishing TN vs. non-TN in the validation cohort. (**A)** ROC curve of the clinical model (blue dotted line) and combined model (red solid line). The AUC of the combined model was 0.868 and that of the clinical model was 0.665. The two ROC curves showed significant difference (p = 0.0449). (**B)** Calibration curves of clinical and combined models. The 45◦ black dotted line expresses the ideal prediction. The combined model is closer to the ideal prediction compared to the clinical model, especially at predicted probability of 0.3 or higher. (**C)** Decision curve of clinical and combined models. In the interval between 5 and 71% of threshold probability, the combined model adds more benefit than applying all or none of the patients, and clinical model.

The multivariate analysis of independent clinical features with the radiomics signature revealed that the radiomics signature was the only statistically significant variable. The combined model yield an AUC value of 0.868 in the validation cohort (Table 5). In the ROC analysis, the performance of the combined model was significantly higher than the clinical model (p = 0.0449, Fig. 4A).

The calibration curve (Fig. 4B) revealed that the combined model demonstrated better agreement between the predicted probability and the expected probability than the clinical model. The clinical decision curve (Fig. 4C) shows that in the threshold probability is 5% or more, the combined model demonstrated a larger net benefit than did the clinical model, indicating that the combined model had the best clinical utility for prediction of TN subtype of breast cancer. The results of the univariate and multivariate analysis for the HER2 and luminal subtype are presented in the supplementary materials (Supplementary Table 4 and 5). The HER2 and luminal subtype did not differ when the performances of the clinical and the combined models were compared in the validation cohort (Table 5).

### Correlation between the radiomics signature and BI-RADS features

**Figure 5:**
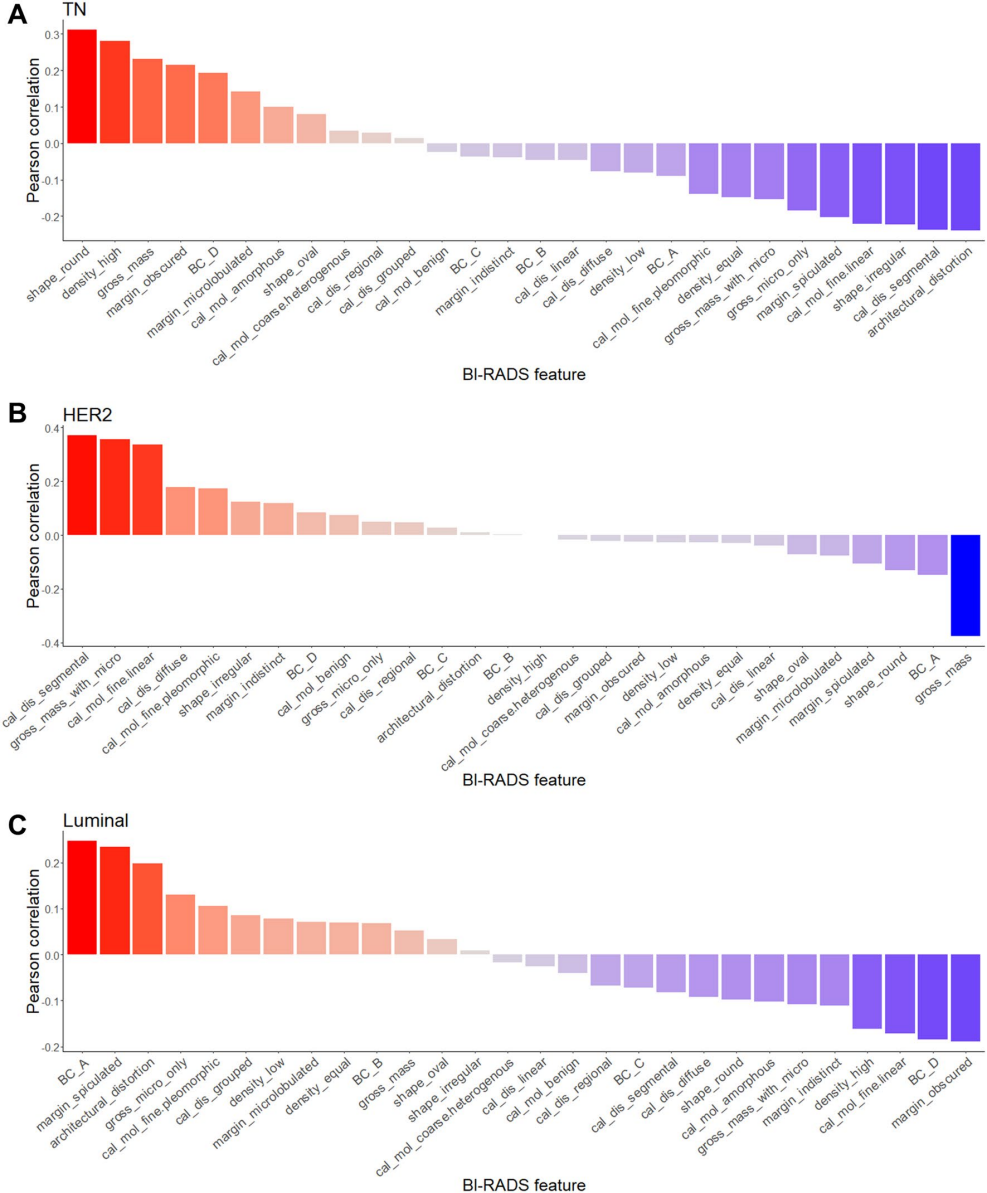
Correlation between the radiomics signature and BI-RADS features for the (**A)** TN, (**B)** HER2 and (**C)** luminal subtype.

The correlations between the radiomics signature and the BI-RADS features for each molecular subtype of breast cancer are shown in Fig. 5 in the order of the correlation coefficient. For the TN subtype, round shape and high density showed a high positive correlation with the radiomics signature. Architectural distortion and segmental distribution of microcalcifications showed negative correlation. For the HER2 subtype, segmental distribution of microcalcifications, mass with microcalcifications and fine linear microcalcifications showed positive correlation with the radiomics signature, and gross features of the mass showed negative correlation. For the luminal subtype, fatty breast composition and spiculated margins showed positive correlation, and obscured margins and dense breast composition showed negative correlation.

## Discussion

This study revealed that the TN subtype of breast cancer can be distinguished by radiomics analysis of synthetic mammography reconstructed from DBT. The radiomics model showed good performance for identifying the TN subtype in the temporally independent validation cohort. In addition, the combined model—a combination of the clinical model and radiomics signature—showed significantly higher performance compared to the clinical model only. This means that the radiomics signature has additive value to the clinical model, which consists of patient age, tumor size and qualitative imaging findings.

The combination of DBT and digital mammography has shown higher sensitivity for breast cancer than digital mammography alone in screening settings^19,20^. However, patients who undergo mammography and DBT at the same time are exposed to higher radiation doses. Thus, efforts have been made to replace digital mammography with synthetic mammography from DBT^21^. Since synthetic mammography from DBT has shown comparable sensitivity with digital mammography, attempts have been made to use DBT alone as a screening modality in North America^22,23^. As the role of DBT increases, more research has been actively conducted on applying radiomics to DBT.

A previous study demonstrated that radiomics could be used in DBT to discriminate cancerous breasts in patients with dense breasts and negative mammography^39^. Another study showed that Ki-67 expression could be predicted using radiomics in DBT^40^. Although these were preliminary results, they suggest that the radiomics methodology can be applied to DBT, similar to mammography. In this study, by using a radiomics analysis of synthetic mammography from DBT, we could discriminate the TN subtype with high performance. Patients with the TN subtype require different treatment approaches such as neoadjuvant chemotherapy for breast cancer than patients with other subtypes due to the absence of targeted agents and poorer prognosis^7^. If we can obtain information about the TN subtype from the screening modality, DBT using radiomics will help clinicians establish appropriate treatment plans. In addition, radiologists can diagnose the TN subtypes with more confidence using the radiomics approach for DBT.

Previous studies using radiomics to predict the molecular subtypes of breast cancer were focused on MRI^14–16^ because of its high soft tissue contrast and visualization of tumor perfusion dynamics. One study reported an overall accuracy of 71.2% for subtyping using only the radiomics features of MRI, and 89.2% when combining these features with pathological features^14^. Another study reported an AUC of 0.65–0.89 for each subtype when using MRI data in TCGA/TCIA^15^. However, these studies only performed internal validation using the leave-one-out method without an independent validation set. Due to differences in the biological characteristics and treatments of TN subtypes, some studies have attempted to distinguish the TN subtype from other subtypes. Radiomics analysis of both tumor and background parenchymal enhancement has increased the AUC from 0.782 to 0.878 when predicting the TN subtype^16^. Although MRI-based radiomics shows high performance, the importance of mammography-based radiomics remains valid, because MRI is an expensive modality and less available than mammography. Meanwhile, mammography is a first-line imaging modality for cancer screening and is applicable to almost all breast cancer patients. Another advantage of mammography is its higher spatial resolution and better ability to visualize microcalcifications compared to MRI. In addition, since mammography can be repeatedly performed during follow-up, it is expected that changes in the molecular subtype that occur frequently after neoadjuvant chemotherapy^41^ will be identified by mammography-based radiomics analysis.

Recent pioneering studies suggested the possibility of predicting molecular subtypes by analyzing digital mammography with radiomics^17,18^. Ma et al. showed that the TN, HER2, and luminal subtype can be distinguished with relatively high performance, and that the discrimination of the TN subtype shows the best performance^17^. Zhang et al. also reported a high performance for distinguishing the TN subtype from non-TN subtypes using radiomics in digital mammography^18^. However, these studies, like many other radiomics studies, are limited in that they did not evaluate an independent validation set. In addition, these studies were performed with digital mammography and it is not known whether the same performance can be guaranteed with synthetic mammography from DBT. The present study showed that a radiomics analysis of synthetic mammography could predict the TN subtype with high performance and validated this higher performance in an independent cohort. Our results showed similar performance levels with previous MRI radiomics studies^16^. The relatively high performance of DBT may be due to the higher resolution of the modality and uniformity of the imaging equipment compared to MRI. MRI has a variety of vendors, image sequences, and numerous image parameters, while DBT only has a limited number of devices commercially available and relatively few parameters, resulting in consistent images. In the study of radiomics, normalization is commonly used to overcome variations in imaging, but the uniformity of DBT equipment itself is still thought to be helpful. Future studies need to be conducted to confirm the multivendor reproducibility of DBT.

In our study, synthetic mammography was used instead of the original DBT image. This approach was chosen to consider actual clinical practice. It is possible to draw ROIs on synthetic mammography using the results of this study in clinical practice. However, it is impractical to draw ROIs on original DBT images. Also, there will be limitations on the reproducibility of ROI on the original DBT images. However, there is the possibility that some tomographic data may be lost on synthetic mammography. Therefore, future research needs to compare synthetic mammography and original DBT images by radiomics analysis.

Because radiomics extract features inherent to an image, correlations between radiomics features and qualitative imaging findings are expected. Several studies have reported that some mammographic findings are associated with certain molecular subtypes of breast cancer^42,43^. The TN subtype of breast cancer has been associated with round or oval mass and circumscribed margin^42^ or oval shaped hyperdense mass^43^. Consistent with these studies, in the present study, round shape and high density showed a high positive correlation with the radiomics signature for predicting the TN subtype. The HER2 subtype was reported to have indistinct margins with suspicious microcalcifications^42^ and the luminal subtype was reported to have spiculated margins and architectural distortion^42^. Similar correlations were found between the radiomics signature and the imaging findings in this study. This means that the radiomics signature reflected mammographic findings associated with each molecular subtype. Conversely, this result means that new imaging findings can be found intuitively through morphological features represented by a combination of features revealed through radiomics analysis. For example, in this study, the radiomics signature suggesting the TN subtype showed positive correlation with obscured or microlobulated margins. Therefore, this needs to be verified in future studies that explore the correlation between mammographic finding and breast cancer subtype.

When trying to distinguish the HER2 and luminal subtype of breast cancer, the radiomics models failed to show sufficient performance in validation. In addition, there was no added value of combining the radiomics signature with the clinical model. When predicting the HER2 and luminal subtype of breast cancer, the radiomics model appeared to be overfitted to the training set and showed inferior performance in the validation cohort. This means that, unlike the TN type, radiomics failed to extract general characteristics suitable for the HER2 and the luminal subtypes. A previous study reported that the phenotypes of the HER2 and luminal subtype had much in common^44^. Microcalcifications and mammographically non-visible masses are well-known common morphologic characteristics of the two subtypes. Therefore, this result may not be a methodological limitation of radiomics, but may actually be due to a classification limitation based on the morphology difference between the HER2 and luminal subtype.

There are several limitations in this study. First, there were inherent limitations due to its retrospective study design. Second, a relatively large number of radiomics features were included in the final model. This makes it difficult to interpret the meaning of each individual radiomics feature. By showing the relationship between the radiomics signature and mammographic features, we verified that mammographic findings were reflected in the molecular subtype predicted by the radiomics analysis. Third, features with Laplacian of Gaussian or wavelet filter were not included. These features can characterize the high-dimensional image signal of the tumor. However, in this study, these features were excluded in consideration of the limited sample size. Future studies with larger sample sizes will need to include an analysis of these features. Fourth, the extraction of radiomics features was based on manually drawn ROIs. To overcome this, features with poor interobserver reproducibility were excluded from the analysis. Fifth, the sample size of the validation cohort is relatively small due to the temporal validation method adopted to split data. In future research, this can be overcome by using a larger sample size or by considering a different data composition method. Sixth, we adopted the binary classification method to classify the three molecular subtypes. This is the method used by existing studies^17,35^, and was intended to obtain intuitive results. In future research, we believe that it is necessary to perform multiclass classification using a different strategy such as softmax. Another limitation was that we only included lesions that were clearly delineated in synthetic mammography. Because the lesion contrast of synthetic mammography was limited compared to the original DBT images, a relatively large number of lesions were excluded from the analysis.

## Conclusions

In conclusion, this study showed a significant relationship between radiomics signatures based on synthetic mammography reconstructed from DBT images and molecular subtypes of breast cancer. The radiomics signature was able to distinguish the TN subtype of breast cancer with high accuracy. Since DBT is an imaging modality that can be performed in almost all patients, the radiomics signature can be used as a potential biomarker for the clinical diagnosis and treatment of breast cancer patients.

## Supplementary Information

Supplementary Information.

## Data Availability

The datasets used and/or analysed during the current study are available from the corresponding author on reasonable request.

## References

[1] Siegel R, Ma J, Zou Z, Jemal A (2014). Cancer statistics, 2014. CA Cancer J. Clin..

[2] Carey LA (2006). Race, breast cancer subtypes, and survival in the Carolina Breast Cancer Study. JAMA.

[3] Goldhirsch, A. *et al.* Strategies for subtypes-dealing with the diversity of breast cancer: highlights of the St. Gallen International Expert Consensus on the Primary Therapy of Early Breast Cancer 2011. *Ann. Oncol.* **22**, 1736–1747 (2011). 10.1093/annonc/mdr304 PMC3144634 21709140

[4] Lam SW, Jimenez CR, Boven E (2014). Breast cancer classification by proteomic technologies: current state of knowledge. Cancer Treat. Rev..

[5] Huber KE, Carey LA, Wazer DE (2009). Breast cancer molecular subtypes in patients with locally advanced disease: impact on prognosis, patterns of recurrence, and response to therapy. Semin. Radiat. Oncol..

[6] Metzger-Filho O (2013). Patterns of recurrence and outcome according to breast cancer subtypes in lymph node-negative disease: Results from international breast cancer study group trials VIII and IX. J. Clin. Oncol..

[7] Liedtke C (2008). Response to neoadjuvant therapy and long-term survival in patients with triple-negative breast cancer. J. Clin. Oncol..

[8] Foulkes WD, Smith IE, Reis-Filho JS (2010). Triple-negative breast cancer. N. Engl. J. Med..

[9] Silver DP (2010). Efficacy of neoadjuvant cisplatin in triple-negative breast cancer. J. Clin. Oncol..

[10] Wu M, Ma J (2017). Association between imaging characteristics and different molecular subtypes of breast cancer. Acad. Radiol..

[11] Celebi F (2015). The role of ultrasonographic findings to predict molecular subtype, histologic grade, and hormone receptor status of breast cancer. Diagn. Interv. Radiol..

[12] Uematsu T, Kasami M, Yuen S (2009). Triple-negative breast cancer: Correlation between MR imaging and pathologic findings. Radiology.

[13] Gillies RJ, Kinahan PE, Hricak H (2016). Radiomics: Images are more than pictures, they are data. Radiology.

[14] Sutton EJ (2016). Breast cancer molecular subtype classifier that incorporates MRI features. J. Magn. Reson. Imaging.

[15] Li, H. *et al.* Quantitative MRI radiomics in the prediction of molecular classifications of breast cancer subtypes in the TCGA/TCIA data set. *NPJ Breast Cancer* **2** (2016). 10.1038/npjbcancer.2016.12 PMC5108580 27853751

[16] Wang J (2015). Identifying triple-negative breast cancer using background parenchymal enhancement heterogeneity on dynamic contrast-enhanced MRI: A pilot radiomics study. PLoS ONE.

[17] Ma W (2019). Breast cancer molecular subtype prediction by mammographic radiomic features. Acad. Radiol..

[18] Zhang HX, Sun ZQ, Cheng YG, Mao GQ (2019). A pilot study of radiomics technology based on X-ray mammography in patients with triple-negative breast cancer. J. Xray Sci. Technol..

[19] Skaane P (2013). Comparison of digital mammography alone and digital mammography plus tomosynthesis in a population-based screening program. Radiology.

[20] Bernardi D (2016). Breast cancer screening with tomosynthesis (3D mammography) with acquired or synthetic 2D mammography compared with 2D mammography alone (STORM-2): A population-based prospective study. Lancet Oncol..

[21] Olgar T, Kahn T, Gosch D (2012). Average glandular dose in digital mammography and breast tomosynthesis. Rofo.

[22] Skaane P (2014). Two-view digital breast tomosynthesis screening with synthetically reconstructed projection images: Comparison with digital breast tomosynthesis with full-field digital mammographic images. Radiology.

[23] Zuckerman SP (2016). Implementation of synthesized two-dimensional mammography in a population-based digital breast tomosynthesis screening program. Radiology.

[24] Tagliafico AS (2017). Accuracy and reading time for six strategies using digital breast tomosynthesis in women with mammographically negative dense breasts. Eur. Radiol..

[25] Howlader, N. *et al.* US incidence of breast cancer subtypes defined by joint hormone receptor and HER2 status. *J Natl Cancer Inst* **106** (2014). 10.1093/jnci/dju055 PMC4580552 24777111

[26] Wei Q, Dunbrack RL (2013). The role of balanced training and testing data sets for binary classifiers in bioinformatics. PLoS ONE.

[27] Yasaka K, Akai H, Abe O, Kiryu S (2018). Deep learning with convolutional neural network for differentiation of liver masses at dynamic contrast-enhanced CT: A preliminary study. Radiology.

[28] Moons KG (2015). Transparent reporting of a multivariable prediction model for individual prognosis or diagnosis (TRIPOD): Explanation and elaboration. Ann. Intern. Med..

[29] Hammond ME, Hayes DF, Wolff AC, Mangu PB, Temin S (2010). American society of clinical oncology/college of american pathologists guideline recommendations for immunohistochemical testing of estrogen and progesterone receptors in breast cancer. J. Oncol. Pract..

[30] Mackin D (2017). Harmonizing the pixel size in retrospective computed tomography radiomics studies. PLoS ONE.

[31] van Griethuysen JJM (2017). Computational radiomics system to decode the radiographic phenotype. Cancer Res.

[32] Zou H, Hastie T (2005). Regularization and variable selection via the elastic net. J. R. Stat. Soc. Ser. B (Stat. Methodol.).

[33] Friedman J, Hastie T, Tibshirani R (2010). Regularization paths for generalized linear models via coordinate descent. J. Stat. Softw..

[34] R Core Team. *R: A Language and Environment for Statistical Computing*. (R Foundation for Statistical Computing, Vienna, 2016). https://www.R-project.org/.

[35] Park HJ (2019). Radiomics analysis of gadoxetic acid-enhanced MRI for staging liver fibrosis. Radiology.

[36] Chawla NV, Bowyer KW, Hall LO, Kegelmeyer WP (2002). SMOTE: Synthetic minority over-sampling technique. J. Artif. Intell. Res..

[37] Blagus R, Lusa L (2013). SMOTE for high-dimensional class-imbalanced data. BMC Bioinform..

[38] D’Orsi, C. J., Sickles, E. A., Mendelson, E. B. & Morris, E. A. *2013 ACR BI-RADS Atlas: Breast Imaging Reporting and Data System* (American College of Radiology, 2014).

[39] Tagliafico AS (2018). An exploratory radiomics analysis on digital breast tomosynthesis in women with mammographically negative dense breasts. Breast.

[40] Tagliafico AS (2019). Breast cancer Ki-67 expression prediction by digital breast tomosynthesis radiomics features. Eur. Radiol. Exp..

[41] Lim SK (2016). Impact of molecular subtype conversion of breast cancers after neoadjuvant chemotherapy on clinical outcome. Cancer Res. Treat..

[42] Boisserie-Lacroix M (2013). Correlation between imaging and molecular classification of breast cancers. Diagn. Interv. Imaging.

[43] Kim MY, Choi N (2013). Mammographic and ultrasonographic features of triple-negative breast cancer: A comparison with other breast cancer subtypes. Acta Radiol..

[44] Ko ES (2010). Triple-negative breast cancer: Correlation between imaging and pathological findings. Eur. Radiol..

