# Supplementary material for: Prediction of breast cancer molecular subtypes using radiomics signatures of synthetic mammography from digital breast tomosynthesis
Source: Sci Rep. 2020 Dec 9;10:21566. doi: 10.1038/s41598-020-78681-9 (PMC7726048; doi:10.1038/s41598-020-78681-9)

Prediction of breast cancer molecular subtypes using radiomics signatures of synthetic mammography from digital breast tomosynthesis

Jinwoo Son, MD, Si Eun Lee, MD, Eun-Kyung Kim, MD, PhD, Sungwon Kim, MD

Supplementary Table 1. List of all features included in this study.

Class	Features	Class	Features
First order	10Percentile	GLRLM	GrayLevelNonUniformity
	90Percentile		GrayLevelNonUniformityNormalized
	Energy		GrayLevelVariance
	Entropy		HighGrayLevelRunEmphasis
	InterquartileRange		LongRunEmphasis
	Kurtosis		LongRunHighGrayLevelEmphasis
	Maximum		LongRunLowGrayLevelEmphasis
	MeanAbsoluteDeviation		LowGrayLevelRunEmphasis
	Mean		RunEntropy
	Median		RunLengthNonUniformity
	Minimum		RunLengthNonUniformityNormalized
	Range		RunPercentage
	RobustMeanAbsoluteDeviation		RunVariance
	RootMeanSquared		ShortRunEmphasis
	Skewness		ShortRunHighGrayLevelEmphasis
	TotalEnergy		ShortRunLowGrayLevelEmphasis
	Uniformity		
	Variance		
GLCM	Autocorrelation	GLSZM	GrayLevelNonUniformity
	JointAverage		GrayLevelNonUniformityNormalized
	ClusterProinence		GrayLevelVariance
	ClusterShade		HighGrayLevelZoneEmphasis
	ClusterTendency		LargeAreaEmphasis
	Contrast		LargeAreaHighGrayLevelEmphasis
	Correlation		LargeAreaLowGrayLevelEmphasis
	DifferenceAverage		LowGrayLevelZoneEmphasis
	DifferenceEntropy		SizeZoneNonUniformity
	DifferenceVariance		SizeZoneNonUniformityNormalized
	JointEnergy		SmallAreaEmphasis
	JointEntropy		SmallAreaHighGrayLevelEmphasis
	Imc1		SmallAreaLowGrayLevelEmphasis
	Imc2		ZoneEntropy
	Idm		ZonePercentage
	Idmn		ZoneVariance
	Id		
	Idn		
	InverseVariance		
	MaximumProbability		
	SumEntropy		
	SumSquares		

GLCM: gray-level co-occurrence matrix, GLRLM: grey-level run length matrix, GLSZM: gray-level size zone matrix

Supplementary Table 2. Lesion size, shape and receptor expression of breast cancers

	TN			HER2			Luminal		
	Training set (N=50)	Validation set (N=12)	P value	Training set (N=50)	Validation set (N=9)	P value	Training set (N=50)	Validation set (N=50)	P value
Lesion size (mm)*	33.98 ± 17.45	29.47 ± 15.04	0.894	41.78 ± 19.55	28.33 ± 10.17	0.050	24.92 ± 14.41	28.68 ± 14.12	0.191
Lesion shape			0.421			0.012			0.176
Oval	3	1		1	3		3	5	
Round	17	2		4	1		8	4	
Irregular	30	9		41	4		36	41	
Non-mass	0	0		4	1		3	0	
ER (percentage)*	0	0	1.000	0	0	1.000	85.34 ± 24.40	82.40 ± 26.46	0.761
PR (percentage)*	0	0	1.000	0	0	1.000	46.14 ± 40.80	50.48 ± 38.60	0.556
HER2			1.000			1.000			0.627
0	25	6		0	0		5	8	
1+	14	3		0	0		20	15	
2+	11	3		10	2		21	24	
3+	0	0		40	7		4	3	

* means ± standard deviations

ER: estrogen receptor, PR: progesterone receptor, HER2: human epidermal growth factor receptor 2

Supplementary Table 3. List of features selected for each radiomics model

Prediction	Selected features
TN vs non-TN	MLO_ firstorder_10Percentile<br>MLO_ firstorder_Entropy<br>MLO_ firstorder_MeanAbsoluteDeviation<br>MLO_ glcm_SumEntropy<br>MLO_ glrlm_GrayLevelNonUniformityNormalized<br>MLO_ glrlm_RunEntropy<br>MLO_ glszm_GrayLevelNonUniformityNormalized<br>CC_ firstorder_10Percentile<br>CC_ firstorder_Entropy<br>CC_ firstorder_MeanAbsoluteDeviation<br>CC_ firstorder_Median<br>CC_ firstorder_Minimum<br>CC_ firstorder_Uniformity<br>CC_ firstorder_Variance<br>CC_ glcm_ClusterTendency<br>CC_ glcm_SumSquares<br>CC_ glrlm_GrayLevelNonUniformityNormalized<br>CC_ glrlm_GrayLevelVariance<br>CC_ glszm_GrayLevelNonUniformityNormalized<br>CC_ glszm_GrayLevelVariance
HER2 vs non-HER2	MLO_firstorder_InterquartileRange<br>MLO_firstorder_Kurtosis<br>MLO_firstorder_Minimum<br>MLO_firstorder_RobustMeanAbsoluteDeviation<br>MLO_glcm_ClusterProminence<br>MLO_glcm_Idn<br>MLO_glszm_GrayLevelNonUniformityNormalized<br>MLO_glszm_LargeAreaHighGrayLevelEmphasis<br>MLO_glszm_LowGrayLevelZoneEmphasis<br>MLO_glszm_SizeZoneNonUniformity<br>MLO_glszm_ZoneEntropy<br>CC_firstorder_Kurtosis<br>CC_firstorder_Minimum<br>CC_glcm_JointAverage<br>CC_glcm_ClusterTendency<br>CC_glcm_Imc2<br>CC_glszm_LargeAreaEmphasis<br>CC_glszm_SizeZoneNonUniformity
Luminal vs non-luminal	MLO_firstorder_10Percentile<br>MLO_firstorder_Entropy<br>MLO_firstorder_InterquartileRange<br>MLO_firstorder_Kurtosis<br>MLO_firstorder_Mean<br>MLO_firstorder_Range<br>MLO_firstorder_RootMeanSquared<br>MLO_firstorder_Skewness<br>MLO_firstorder_Variance<br>MLO_glcm_JointAverage<br>MLO_glcm_ClusterShade<br>MLO_glcm_ClusterTendency<br>MLO_glcm_DifferenceVariance<br>MLO_glcm_Idm<br>MLO_glcm_Idmn<br>MLO_glcm_Id<br>MLO_glcm_InverseVariance<br>MLO_glcm_SumEntropy<br>MLO_glcm_SumSquares<br>MLO_glrlm_GrayLevelNonUniformity<br>MLO_glrlm_LongRunLowGrayLevelEmphasis<br>MLO_glrlm_LowGrayLevelRunEmphasis<br>MLO_glrlm_RunLengthNonUniformityNormalized<br>MLO_glrlm_RunPercentage<br>MLO_glrlm_ShortRunEmphasis<br>MLO_glrlm_ShortRunLowGrayLevelEmphasis<br>MLO_glszm_HighGrayLevelZoneEmphasis<br>MLO_glszm_LargeAreaLowGrayLevelEmphasis<br>MLO_glszm_LowGrayLevelZoneEmphasis<br>MLO_glszm_SmallAreaHighGrayLevelEmphasis<br>MLO_glszm_SmallAreaLowGrayLevelEmphasis<br>MLO_glszm_ZoneEntropy<br>MLO_glszm_ZonePercentage<br>CC_firstorder_10Percentile<br>CC_firstorder_Entropy<br>CC_firstorder_Kurtosis<br>CC_firstorder_Maximum<br>CC_firstorder_MeanAbsoluteDeviation<br>CC_firstorder_Mean<br>CC_firstorder_Median<br>CC_firstorder_Minimum<br>CC_firstorder_Range

	CC_firstorder_RootMeanSquared
	CC_firstorder_Skewness
	CC_firstorder_Uniformity
	CC_firstorder_Variance
	CC_glcm_Autocorrelation
	CC_glcm_ClusterShade
	CC_glcm_ClusterTendency
	CC_glcm_Correlation
	CC_glcm_Imc2
	CC_glcm_SumSquares
	CC_glrlm_GrayLevelNonUniformity
	CC_glrlm_GrayLevelNonUniformityNormalized
	CC_glrlm_GrayLevelVariance
	CC_glrlm_HighGrayLevelRunEmphasis
	CC_glrlm_RunLengthNonUniformity
	CC_glrlm_ShortRunHighGrayLevelEmphasis
	CC_glszm_GrayLevelNonUniformity
	CC_glszm_HighGrayLevelZoneEmphasis
	CC_glszm_LargeAreaEmphasis
	CC_glszm_LargeAreaHighGrayLevelEmphasis
	CC_glszm_SizeZoneNonUniformity
	CC_glszm_SmallAreaEmphasis
	CC_glszm_SmallAreaHighGrayLevelEmphasis
	CC_glszm_ZoneVariance

CC: craniocaudal, MLO: mediolateral oblique, GLCM: gray-level co-occurrence matrix, GLRLM: grey-level run length matrix, GLSZM: gray-level size zone matrix

Supplementary Table 4. Univariate and multivariate logistic regression of the clinical model and combined model for the HER2 subtype of breast cancer.

			Univariate analysis		Multivariate analysis		With the radiomics signature	
Feature	HER2	Non-HER2	P value	Odds ratio	P value	Odds ratio	P value	Odds ratio
Age	52.70 ± 8.510	54.87 ± 10.69	0.213	0.978 (0.944, 1.012)				
Size	41.78 ± 19.55	29.45 ± 16.56	<0.001	1.037 (1.018, 1.059)	0.036	1.024 (1.002, 1.048)	0.382	0.987 (0.956, 1.016)
Breast composition								
Dense	41	70	Ref	1				
Fatty	9	30	0.118	0.512 (0.211, 1.149)				
Gross features								
Mass only	16	59	Ref	1				
Mass + calcification	30	38	0.004	2.911 (1.418, 6.157)	0.991	NA	0.993	NA
Calcification only	4	3	0.050	4.917 (0.990, 27.170)				
Shape								
Oval	1	6	0.231	0.268 (0.014, 1.648)				
Round	4	25	0.018	0.258 (0.072, 0.722)	0.081	0.352 (0.095, 1.047)	0.033	0.208 (0.042, 0.795)
Irregular	41	66	Ref	1				
Mass margin								
Obscured	12	16	0.515	1.339 (0.548, 3.225)				
Microlobulated	3	13	0.194	0.412 (0.089, 1.412)				
Indistinct	28	50	Ref	1				
Spiculated	3	18	0.069	0.298 (0.066, 0.977)				
Mass density								
Low	1	9	0.102	0.172 (0.009, 0.977)				
Equal	33	51	Ref	1				
High	12	37	0.084	0.501 (0.222, 1.079)				
Architectural distortion	7	23	0.198	0.545 (0.202, 1.318)				
Calcification morphology								
Benign	0	2	0.988	NA				
Amorphous	1	3	0.541	0.481 (0.023, 4.107)				
Coarse heterogeneous	3	5	0.857	0.867 (0.161, 3.995)				
Fine pleomorphic	18	26	Ref	1				
Fine linear branching	13	5	0.030	3.756 (1.192, 13.452)	0.130	2.707 (0.773, 10.604)	0.371	2.015 (0.447, 9.978)
Calcification distribution								
Diffuse	1	0	0.995	NA				
Regional	0	4	0.989	NA				
Grouped	5	12	0.076	0.345 (0.098, 1.070)				
Linear	0	1	0.994	NA				
Segmental	29	24	Ref	1				
Radiomics signature			<0.001	283 (50, 2140)			<0.001	616 (61, 9168)

Supplementary Table 5. Univariate and multivariate logistic regression of the clinical model and combined model for the luminal subtype of breast cancer.

			Univariate analysis		Multivariate analysis		With radiomics signature	
Feature	Luminal	Non-Luminal	P value	Odds ratio	P value	Odds ratio	P value	Odds ratio
Age	55.66 ± 10.95	53.39 ± 9.520	0.193	1.023 (0.989, 1.059)				
Size	24.92 ± 14.41	37.88 ± 18.85	<0.001	0.947 (0.919, 0.972)	0.002	0.946 (0.191, 0.977)	0.864	0.996 (0.946, 1.044)
Breast composition								
Dense	30	81	Ref	1				
Fatty	20	19	0.007	2.842 (1.339, 6.098)	0.014	3.289 (1.291, 8.708)	0.341	1.996 (0.472, 8.487)
Gross feature								
Mass only	30	45	Ref	1				
Mass + calcification	17	51	0.058	0.500 (0.240, 1.015)				
Calcification only	3	4	0.883	1.125 (0.209, 5.454)				
Shape								
Oval	3	4	0.621	1.479 (0.279, 7.057)				
Round	8	21	0.537	0.751 (0.289, 1.809)				
Irregular	36	71	Ref	1				
Mass margin								
Obscured	6	22	0.487	0.694 (0.231, 1.861)				
Microlobulated	6	10	0.461	1.527 (0.471, 4.638)				
Indistinct	22	56	Ref	1				
Spiculated	13	8	0.006	4.136 (1.535, 11.794)	0.244	2.344 (0.544, 10.011)	0.470	2.243 (0.229, 2.045)
Mass density								
Low	6	4	0.109	3.000 (0.793, 12.560)				
Equal	28	56	Ref	1				
High	13	36	0.413	0.722 (0.324, 1.555)				
Architectural distortion	18	12	0.001	4.125 (1.810, 9.724)	0.007	5.577 (1.663, 20.503)	0.266	3.250 (0.448, 29.423)
Calcification morphology								
Benign	1	1	0.649	1.933 (0.073, 51.238)				
Amorphous	1	3	0.714	0.644 (0.030, 5.538)				
Coarse heterogeneous	2	6	0.616	0.644 (0.087, 3.204)				
Fine pleomorphic	15	29	Ref	1				
Fine linear branching	1	17	0.044	0.114 (0.006, 0.640)	0.198	0.167 (0.005, 1.688)	0.458	0.298 (0.006, 4.625)
Calcification distribution								
Diffuse	0	1	0.992	NA				
Regional	2	2	0.169	4.300 (0.471, 39.595)				
Grouped	8	9	0.025	3.822 (1.178, 12.664)	0.443	1.794 (0.403, 8.268)	0.166	4.310 (0.578, 38.452)
Linear	0	1	0.992	NA				
Segmental	10	43	Ref	1				
Radiomics signature			<0.001	1536 (221, 17693)			<0.001	1673 (170, 3099)

Supplementary Figure 1. Interobserver reproducibility for each radiomics feature was shown with the intraclass coefficient (ICC) for the A. MLO view and B. CC view. An ICC > 0.75 was considered to indicate good agreement.

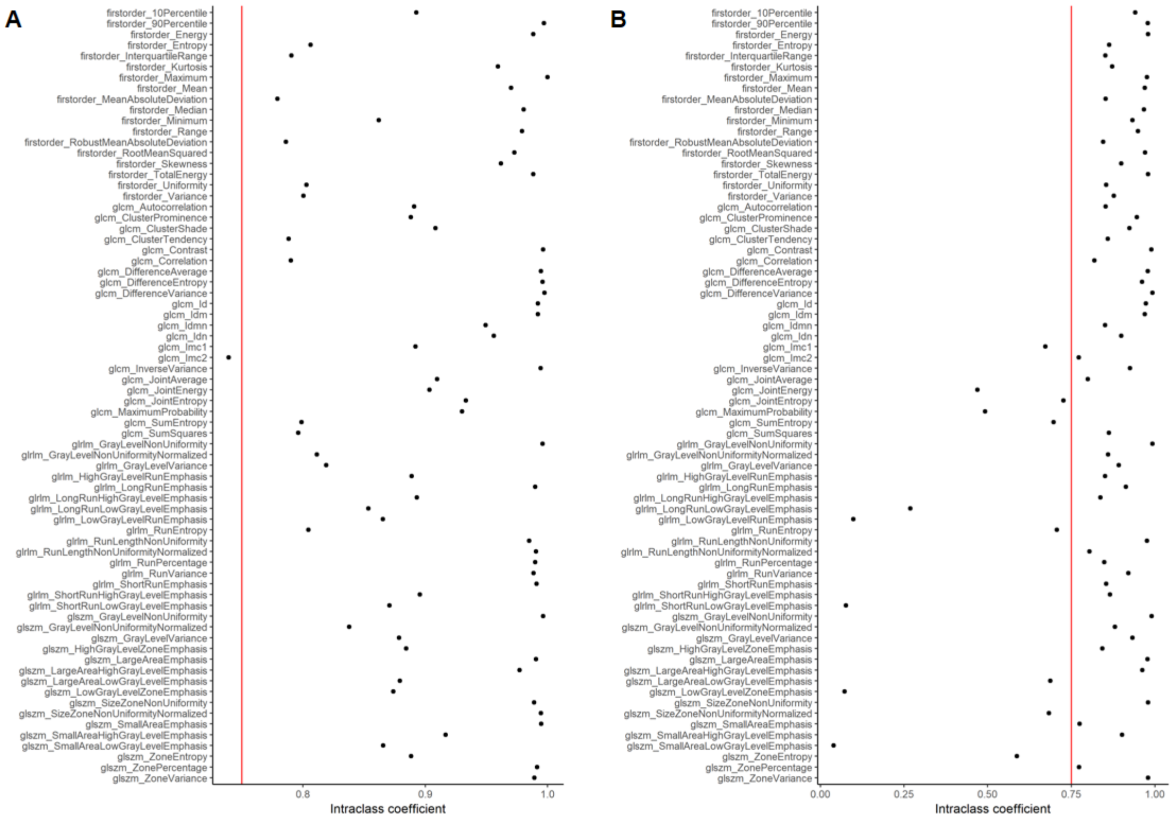

Supplement: Supplementary file 1 — Supplementary Information. [file 41598_2020_78681_MOESM1_ESM.pdf]
